# Pathological Consequences of Hepatic mTORC1 Dysregulation

**DOI:** 10.3390/genes11080896

**Published:** 2020-08-05

**Authors:** Chun-Seok Cho, Allison Ho Kowalsky, Jun Hee Lee

**Affiliations:** 1 Department of Molecular & Integrative Physiology, University of Michigan, Ann Arbor, MI 48109, USA; chochun@umich.edu (C.-S.C.); allho@umich.edu (A.H.K.); 2 Department of Surgery, University of Michigan, Ann Arbor, MI 48109, USA

**Keywords:** mTORC1, liver, metabolism

## Abstract

The mammalian target of rapamycin complex 1 (mTORC1) is a central regulator of metabolism that integrates environmental inputs, including nutrients, growth factors, and stress signals. mTORC1 activation upregulates anabolism of diverse macromolecules, such as proteins, lipids, and nucleic acids, while downregulating autolysosomal catabolism. mTORC1 dysregulation is often found in various diseases, including cancer, cardiovascular and neurodegenerative diseases, as well as metabolic syndromes involving obesity and type II diabetes. As an essential metabolic organ, the liver requires proper regulation of mTORC1 for maintaining homeostasis and preventing pathologies. For instance, aberrant hyper- or hypoactivation of mTORC1 disrupts hepatocellular homeostasis and damages the structural and functional integrity of the tissue, leading to prominent liver injury and the development of hepatocellular carcinogenesis. Proper regulation of mTORC1 during liver diseases may be beneficial for restoring liver function and ameliorating the detrimental consequences of liver failure.

## 1. Introduction: mTOR Complexes

The mammalian target of rapamycin (mTOR) is a protein kinase, which can be found in two protein complexes, named mTOR complex 1 (mTORC1) and mTORC2 in cells [1]. mTORC1 is the rapamycin-sensitive mTOR complex that upregulates cellular anabolism of proteins, lipids, and nucleic acids, and downregulates autophagic catabolism. p70 ribosomal protein S6 kinase (S6K), eukaryotic translation initiation factor 4E-binding protein 1 (4E-BP1), unc-51-like autophagy activating kinase 1 (ULK1), and transcription factor EB (TFEB) are well-characterized substrates of mTORC1 that mediate its metabolic effects [2]. In contrast, mTORC2 is the rapamycin-insensitive mTOR complex with a different set of substrates, such as AKT, serum and glucocorticoid inducible kinase 1 (SGK1), and protein kinase C (PKC) [3]. In addition to mTORC1 and mTORC2, several other mTOR complexes were recently reported, including GIT1 [4], mEAK-7 [5,6], and ETV7 [7]. In this review, we will focus on the rapamycin-sensitive mTORC1, and its regulation of liver homeostasis and pathophysiology.

## 2. Regulation of mTORC1 by Environmental Inputs

### 2.1. Insulin/Growth Factor-Dependent Control

**Figure 1:**
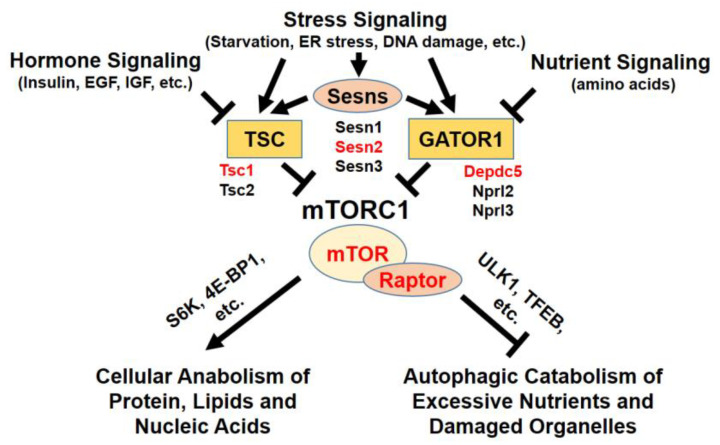
Upstream and downstream of mTORC1 signaling. mTORC1 is regulated by hormone, growth factor, stress, and nutrient signaling. Through signaling mediators, such as Sestrins, TSC, and GATOR1 protein complexes, mTORC1 activity is delicately modulated in cells. Active mTORC1 kinase phosphorylates various substrates to regulate cell metabolism. Red-highlighted genes were mutated in livers to understand the hepatocellular role of mTORC1.

mTORC1 is potently activated by insulin and growth factor signals [8], as well as amino acids [2,9] (Figure 1). Insulin/growth factor-induced mTORC1 activation is mainly dependent on the phosphoinositide 3-kinase (PI3K)–AKT signaling [8]. Once activated by insulin/growth factor signals, AKT phosphorylates and inhibits tuberous sclerosis complex 2 (TSC2) [10] and PRAS40 [11,12], which are negative regulators of mTORC1. TSC2 is an essential component of the TSC GTPase-activating protein (GAP) complex that inhibits Rheb GTPase, which is essential for mTORC1 activation [13]. PRAS40 is an inhibitory subunit of mTORC1 protein kinase [14]. Therefore, both TSC2 and PRAS40 are important mediators of AKT to activate mTORC1.

### 2.2. Energy-Dependent Control

Opposite to insulin and growth factor signals which activate mTORC1, energy deprivation can silence mTORC1 signaling through activation of AMP-activated protein kinase (AMPK) [15]. AMPK senses the levels of AMP, which is accumulated during ATP depletion [16]. Recent studies indicate that AMPK also senses cellular glucose levels independent of AMP [17]. In conditions of ATP depletion or glucose starvation, AMPK is activated and phosphorylates TSC2. In contrast to AKT-dependent inhibitory phosphorylation, AMPK-dependent TSC2 phosphorylation upregulates TSC GAP activity on Rheb; therefore, AMPK inhibits mTORC1 by downregulating Rheb [15]. AMPK can also directly inhibit mTORC1 by phosphorylating its essential subunit Raptor [18]. AMPK-dependent Raptor phosphorylation inhibits mTORC1 catalytic activity towards its main substrates, such as S6K [18]. AMPK-dependent phosphorylation sites on TSC2 and Raptor are evolutionarily conserved in invertebrates [18,19], signifying the importance of this mechanism of regulation.

### 2.3. Amino Acid-Dependent Control

The presence of amino acids is an essential prerequisite for full activation of mTORC1 in response to insulin and growth factors [2,9]. Amino acid signaling is mediated through another GTPase named Rag [9]. Rag is regulated by a heterotrimeric GAP protein complex named GAP activity toward Rags 1 (GATOR1), which has three subunits: DEPDC5, NPRL2, and NPRL3 [20,21,22]. In the presence of amino acids, GATOR1 activity is downregulated, and Rag is subsequently upregulated [22]. In addition, a guanylate exchange factor (GEF) complex named Ragulator also regulates Rag activation upon amino acid stimulation [23]. Multiple molecular sensors for different amino acids were suggested to mediate the amino acid-dependent regulation of GATOR1 and Ragulator [24]. Although Rag is considered generally important for amino acid sensing, recent studies indicate that glutamine and asparagine can upregulate mTORC1 independently of Rag [25,26]. Likewise, leucine can also Rag-independently activate mTORC1 through EP300-mediated acetylation of Raptor [27].

### 2.4. Environmental Stress-Dependent Control

DNA damage is known to suppress mTORC1 in normal instances of DNA double-stranded break [28]; this regulation is important for cell survival by halting cell growth and preserving resources for repair processes. Sestrin1 and Sestrin2 are essential mediators of DNA damage-induced mTORC1 silencing [29]. Sestrins suppress mTORC1 through activation of the AMPK–TSC2 axis [29] and upregulation of the GATOR1 axis [30,31,32,33]. In addition to DNA damage, Sestrins mediate mTORC1 inhibition during other stress conditions such as ER stress [34,35,36,37,38]. Other stress-inducible proteins and structures, such as regulated in development and DNA damage responses 1/2 (REDD1/2) and stress granule proteins also stress-dependently regulate mTORC1 [39].

## 3. Pathological Consequences of mTORC1 Dysregulation in Liver

mTORC1 components, as well as their regulators, were modulated in mouse livers (Figure 1) through molecular genetic tools such as Alb-Cre-mediated conditional modulation. Alb-Cre, which is the tool used for most of the studies described below, deletes the corresponding floxed genetic components in all hepatocytes of various developmental stages, including fetal and neonatal mice; therefore, it is appropriate for examining the genetic component in the development and function of hepatocytes [40].

### 3.1. Ablation of mTOR

Liver-specific deletion of the *mTOR* gene was recently examined. Although the liver developed normally, mTOR-deficient livers displayed mild steatosis associated with upregulation of lipogenic transcription factors *Pparg2* and *Srebf1*, and increased expression of their target genes, including *Acaca* (catalyzes the carboxylation of acetyl-CoA to malonyl-CoA, the rate limiting step in fatty acid synthesis), *Fasn* (catalyzes fatty acid synthesis from acetyl-CoA and malonyl-CoA), *Gpam* (catalyzes the initial step in glycerolipid biosynthesis), and *Dgat1* (catalyzes the formation of triglycerides from diacylglycerol and Acyl-CoA) [41]. Liver-specific mTOR-knockout mice were more sensitive to hepatic ischemia/reperfusion injury [41] through upregulation of pro-inflammatory cytokine signaling.

### 3.2. Ablation of mTORC1

In addition to the mTOR catalytic subunit, mTORC1 contains many regulatory subunits, including Raptor, mLst8, Deptor, and Pras40 (inhibitory subunit). Raptor is the essential subunit of mTORC1, defining its substrate specificity and functionality [42,43,44]. Therefore, liver-specific *Raptor*-knockout mice have been extensively utilized to understand the physiological role of mTORC1 in the liver.

Initially, it was reported that Raptor ablation suppressed liver fat accumulation in response to a high-fat, high-cholesterol, Western diet [45]. The improvement of hepatosteatosis was associated with the inhibition of SREBP1 target gene expression [45]. The liver-specific *Raptor* knockout, however, did not abolish fructose-induced activation of the SREBP1 pathways [46]. In an independent study, liver-specific Raptor ablation unexpectedly developed spontaneous hepatosteatosis in lean mice [47]. This steatosis was independent of compensatory AKT upregulation, due to improved insulin sensitivity, but was dependent on decreased phosphatidylcholine synthesis [47]. Raptor also had mTORC1-independent effects in preventing hepatosteatosis [48]; therefore, the role of Raptor in hepatic fat metabolism seems to be complicated.

Raptor ablation and mTORC1 downregulation interfered with hepatic homeostasis by provoking spontaneous liver damage and pro-inflammatory cytokine signaling [49]. When combined with a hepatocarcinogen diethylnitrosamine (DEN) and a high-fat diet (HFD), liver-specific *Raptor*-knockout mice were more susceptible to liver cancer development and growth [49]. Therefore, although mTORC1 is generally considered a promoter of carcinogenic cell growth, mTORC1 hypoactivity in Raptor-deficient hepatocytes paradoxically promoted the development of hepatocellular carcinoma (HCC) in the DEN-HFD model.

### 3.3. Concurrent Ablation of Both mTORC1 and Autophagy

Autophagy, one of the major outputs of mTORC1 signaling, is important for liver homeostasis [50]. Hepatic autophagy deficiency induces hepatomegaly and inflammation [51], leading to liver tumorigenesis [52]. Interestingly, defective liver autophagy was shown to activate mTORC1, but this mTORC1 upregulation was actually required for pathological progression of autophagy-deficient livers. Specifically, liver-specific *Atg5*-knockout mice suffered severe hepatomegaly and liver inflammation, but these pathologies were relatively attenuated in liver-specific *Atg5*/*mTOR* or *Atg5*/*Raptor* double knockout mice [53]. However, these liver-specific *Atg5*/*mTOR* or *Atg5*/*Raptor* double knockout mice exhibited accelerated liver tumor development compared to the liver-specific *Atg5* single knockout mice [53]. Combined with the results from the HFD model where liver-specific Raptor deletion promoted DEN-induced liver cancer [49], these results demonstrated that mTORC1 hypoactivity can promote carcinogenic processes in the liver.

### 3.4. Upregulation of Insulin/Growth Factor Signaling on mTORC1

As previously mentioned, the PI3K–AKT pathway and Rheb GTPase are critical for mediating insulin/growth factor-induced activation of mTORC1. PTEN is an important negative regulator of PI3K signaling by dephosphorylating phosphatidylinositol (3,4,5)-trisphosphate (PIP3), which is generated by PI3K as a second messenger [54]. PTEN ablation leads to persistent PIP3 levels, leading to hyperactivation of the AKT–Rheb signaling axis [8,54]. TSC is another important negative regulator of this pathway that suppresses Rheb as a GAP [13].

Liver-specific ablation of PTEN and TSC1 showed many interesting phenotypes associated with mTORC1 upregulation. Liver-specific PTEN ablation led to strong steatohepatitis that progressed to HCC development [55,56,57]. Insulin–AKT signaling was hyperactivated in *PTEN*-ablated hepatocytes, leading to increased glucose tolerance and hypoglycemia [55]. In contrast, liver-specific *TSC1* ablation did not induce fat accumulation, but rather inhibited insulin–AKT signaling, likely through a feedback mechanism [58]. *TSC1* ablation in the liver decreased ketone body production [59] and made the liver more resistant to high-fat diet (HFD)-induced hepatosteatosis [58]. Still, like *PTEN*-knockouts, *TSC1* ablation induced HCC development [60]. *TSC1*-knockout livers prominently accumulated autophagy substrate p62/SQSTM1, and p62/SQSTM1 was important for liver damage and hepatocarcinogenesis upon the loss of TSC1 [61].

AKT can also directly activate mTORC1 through PRAS40 inhibition in a TSC-independent manner. Both mTORC1 and AKT signaling pathways were upregulated in the *PRAS40*-knockout mice, leading to improved hepatic insulin sensitivity and glucose tolerance [62].

Double mutation of *PTEN* and *TSC1* resulted in more severe liver pathologies than the single knockouts [63]. Although *Pten* and *Tsc1* single liver-specific knockout mice developed liver cancer after 40 weeks of age, *Pten/Tsc1* double knockout mice developed HCC by 20 weeks. These results supported that Pten and Tsc1 are not redundant carcinogenic regulators of mTORC1; AKT signaling can TSC-independently upregulate mTORC1 signaling [11,12], and TSC had signaling inputs other than PI3K–AKT, such as AMPK and GSK3 [15,64].

Collectively, these results indicated that proper regulation of insulin/growth factor signaling on mTORC1 was critical for suppressing hepatocarcinogenesis and maintaining hepatocellular homeostasis.

### 3.5. Upregulation of Amino Acid Signaling on mTORC1

DEPDC5 is a protein in the GATOR1 complex that upregulates amino acid-dependent Rag signaling [22]. Recently, liver-specific *DEPDC5*-knockout mice were described and analyzed [65]. *DEPDC5*-knockout mice exhibited many phenotypes similar to *Tsc1*-knockout mice, such as increased basal inflammation and resistance to HFD-induced steatosis [65]. Transcriptome analysis of liver-specific single knockout mice of *Tsc1* or *DEPDC5* indeed revealed that they altered the liver transcriptome similarly [65]. Therefore, even though the method of mTORC1 upregulation was different between the liver-specific knockouts of *Tsc1* and *DEPDC5*, consequences of mTORC1 activation appeared to be similar between the two models.

### 3.6. Hyperactivation of mTORC1 through Both Growth Factor and Nutrient Pathways

Even though the liver-specific knockout phenotypes of *Tsc1* and *Depdc5* were similar, they regulated mTORC1 through separate parallel pathways. Concurrent mutation of *Tsc1* and *Depdc5* in the liver produced prominent upregulation of mTORC1, which was not seen in the single knockout mutants [65]. Interestingly, mTORC1 hyperactivation in double knockouts severely injured the liver through a prominent accumulation of oxidative stress in hepatocytes [65]. This led to excessive hepatocellular injury and death, leading to functional liver failure, indicated by the dramatic elevation of serum liver enzymes and bilirubin concentrations [65]. All of these pathologies were suppressed with just 10 days of rapamycin treatment, suggesting that mTORC1 hyperactivation was the sole output of concurrent *Tsc1/Depdc5* mutation into liver damage [65]. In addition, administration of the antioxidant N-acetyl cysteine or superoxide scavenger Tempol also dramatically ameliorated the liver damage in the *Tsc1*/*Depdc5* double knockout mice [65]. These results indicated that mTORC1 hyperactivation injured the liver mainly through induction of oxidative stress and subsequent hepatocellular damage.

### 3.7. Ablation of Stress-Dependent mTORC1 Regulation Mechanisms

Sestrins are important feedback regulators for mTORC1 that respond to various environmental stresses, including nutritional, DNA damage, chemical, and ER stress insults [66,67]. Sestrin2 and other Sestrin paralogs were extensively studied in the liver and found to regulate mTORC1 signaling [31,35,68], as well as other signaling pathways such as mTORC2 [69,70], redox signaling [71], and TGF-β signaling [72]. With regards to mTORC1 regulation, Sestrin2 was shown to be critical for silencing mTORC1 after administration of DEN, a hepatocarcinogen that damages DNA [29]. Sestrin2 was also important for shutting down mTORC1 activity after hepatic ER stress, either induced acutely through a chemical tunicamycin or chronically through HFD-induced obesity and fatty liver [35]. Proper downregulation of mTORC1 by Sestrin2 was important for inducing autophagy of lipid droplets [68] and suppressing unfolded protein stress [35] during dietary and genetic obesity. During obesity, Sestrin2 deficiency subsequently exacerbated ER stress-associated liver pathologies, including fat accumulation, hepatocellular damage, liver fibrosis, and insulin resistance [35,68].

## 4. Conclusions

**Figure 2:**
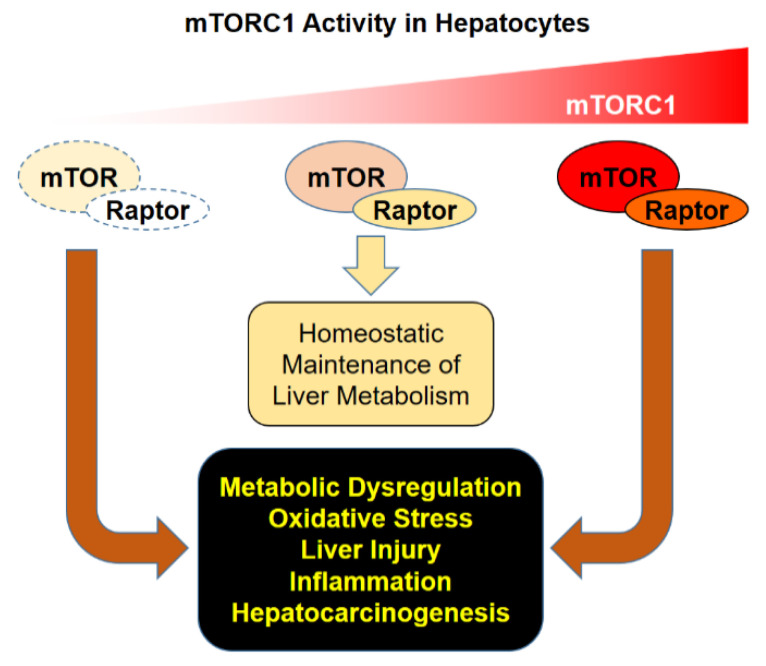
Consequences of mTORC1 dysregulation. Proper regulation of mTORC1 activity is critical for homeostatic maintenance of liver metabolism. Hypo- or hyper-activation of mTORC1 can provoke metabolic dysregulation and oxidative stress, which can lead to hepatocyte injury, inflammation, and subsequent hepatocarcinogenesis.

Proper regulation of mTORC1 activity is critical for maintaining hepatocellular homeostasis and suppressing hepatic injury, inflammation, and carcinogenesis. Aberrant hyper- or hypoactivation of mTORC1 dysregulates hepatocellular homeostasis, resulting in liver damage, inflammation, and subsequent carcinogenesis (Figure 2). Loss of physiological mechanisms that suppress mTORC1 activation, mediated through TSC and GATOR1, can lead to persistent mTORC1 upregulation that is associated with hepatocellular dysfunction, inflammation, and HCC development. However, loss of mTORC1 activity, through loss of mTOR kinase or Raptor, can also promote HCC development in many contexts, including chemical carcinogenesis and autophagy deficiency-induced carcinogenesis. Many functions of mTORC1 in regulating metabolism are also context-specific, as exemplified by the different effects of mTORC1 modulation on hepatic lipid metabolism. The complex role of mTORC1 in regulating liver metabolism, homeostasis, and pathophysiology might be one of the reasons of why different mTORC1 inhibitors were not successful in treating liver cancer in clinical trials [73]. More research is necessary to understand the mechanisms of how mTORC1 dysregulation provokes liver injury and pathologies, which could lead to the development of better mTOR-based therapeutics for various liver diseases.
